# Exploring Factors Influencing Students’ Entrepreneurial Intention in Vocational Colleges Based on Structural Equation Modeling: Evidence From China

**DOI:** 10.3389/fpsyg.2022.898319

**Published:** 2022-06-07

**Authors:** Xiaoqian Fu, Tianming Yan, Yumi Tian, Xinchen Niu, Xin Xu, Yao Wei, Qifan Hu, Zhongming Ouyang, Xueshi Wu

**Affiliations:** ^1^School of Education, Jiangxi Science and Technology Normal University, Nanchang, China; ^2^School of International Education, Zhejiang International Maritime College, Zhoushan, China

**Keywords:** higher vocational college students, entrepreneurial self-efficacy, subjective norms, emotional competency, entrepreneurial education, entrepreneurial attitude, entrepreneurial intention

## Abstract

With the proposal of “mass entrepreneurship, mass innovation” and other ideas, the demand for entrepreneurial talent in China is increasing, but the supply of entrepreneurial talent is far insufficient. Consistent with theory of social cognition and planned behavior, this study outlines a conceptual model including entrepreneurial intention (EI), emotional competency (EC), entrepreneurial self-efficacy (ESE), entrepreneurial attitude (EA), entrepreneurial education (EE), and subjective norms (SN). A structural equation model was applied through a questionnaire survey of 382 vocational college students in Jiangxi province to test the relationship between the constructs in the model. The results show that, firstly, EA, EE, ESE, and EC have positive effects on EI, while the positive effect of SN on EI is not supported. Secondly, a mediating role is played by ESE and EA in the association between EI and EE. Thirdly, ESE and EA play mediating roles in the relationship between EI and EC. Some implications of EI for schools and students were discussed.

## Introduction

In an era of increasing focus on technological progress and strong international competition, entrepreneurship is perceived not only as an approach to boost employment as well as social and political stability but also as a source for competition and innovation (Shane and Venkataraman, 2000). Entrepreneurs are the key to entrepreneurial activities, where entrepreneurial behavior is seen as voluntary and spontaneous, and entrepreneurial education is a tool for training entrepreneurs and enhancing entrepreneurial activities (Frye, 2018). Many studies have supported the predictive validity of intention on actual behavior and have regarded entrepreneurial intention as one of the key prerequisites for entrepreneurs to implement entrepreneurial behavior (Bird, 1988; Ajzen, 1991; Krueger et al., 2000), arguing that entrepreneurial intention can be cultivated (Gnyawali and Fogel, 1994). Young people, such as college students, are more inclined to possess better prospects for enterprise development and succeed in establishing innovative businesses (Liñán, 2008; Padilla-Angulo, 2017). Consequently, many scholars have studied students’ entrepreneurial intention and entrepreneurial education from various perspectives. Among notable research studies, Theory of Planned Behavior (TPB) has been widely employed because of its powerful explanatory and predictive power. On the basis of Theory of Planned Behavior, researchers have added other theoretical perspectives (such as social cognition theory, self-efficacy theory, and emotional intelligence perspective) to adjust Theory of Planned Behavior model to adapt to distinctive economic and cultural backgrounds (Palamida et al., 2018; Mozahem and Adlouni, 2021; Otache et al., 2021). However, in China, such research are insufficient. In 2018, General Secretary Xi Jinping made an important speech at the National Education Conference, calling for entrepreneurial education as well as innovation to go through the entire process of talent training in order to cultivate creative talent through creative education and create an innovative country with creative talent. There are many types of entrepreneurship supporting education, among which the course of higher education level entrepreneurship is an awareness education, specific for zero-experience students, aiming at improving their intention of entrepreneurship and training aspiring entrepreneurs (Liñán et al., 2010; Bae et al., 2014).

Vocational education is a significant attribute of higher education. From the perspective of the high-quality development of vocational education in China, it is vital to run entrepreneurial education well in higher vocational colleges to cater to the development of the times. Studying the current situation of students’ entrepreneurial intention in vocational colleges, providing each student with practical and effective entrepreneurial education with determination and persistence in starting a business, and cultivating their knowledge and skills will not only help students succeed in starting a business but also create opportunities and more value for society.

The purpose of this study is to explore the key driving factors influencing the entrepreneurial intention of vocational college students and the relationship between these factors. After this introduction, this study puts forward the proposed theories and postulated hypotheses and then describes the methods used: sample description, research instrument, data analysis, path analysis, and research results. Finally, this study has drawn some conclusions, highlighted the practical significance, and acknowledged the inherent limitations of this research study.

## Literature Review

### Theory of Planned Behavior

In 1985, Ajzen added perceived behavior control (PBC) variable based on Reasoned Action Theory and preliminarily proposed TPB (Ajzen, 1985). Later, the paper “TPB” was published in 1991, marking the maturity of the theory. According to TPB, all factors that may influence behavior indirectly affect behavior performance through behavioral intention, which is the result of the joint influence of attitude toward behavior (ATB), SN, and PBC. ATB represents the negative or positive feelings of a person regarding an action. The conceptualization of a person’s assessment pertaining to a certain behavior forms such an attitude. SN highlights the social pressure endured by a person when deciding to exhibit a specific behavior. It also shows the effect of salient individuals or social groups, such as relatives and friends on individual behavioral decisions. PBC indicates the extent to which a person feels the difficulty or ease of exercising a certain set of behaviors. It demonstrates the perception of the person related to the factors that hinder or promote executive behavior. ATB, PBC, and SN are conceptually distinct but interrelated, and they work together on behavioral intentions. The more positive a person’s ATB is, the more supported by important people around him, the stronger his PBC is, and the stronger his behavioral intention will be.

TPB has high predictive validity in behavior prediction, which can explain 39 and 27% of variance in intention and behavior, respectively, (Armitage and Conner, 2001). Up till the present moment, TPB is the most widely used theory to explain EI (Fernández-Pérez et al., 2017; Wijayati et al., 2021). TPB model provides a simple model for the main determinants of individual behavior and has been widely concerned with and applied in many research fields, such as social science and entrepreneurial management.

### Entrepreneurial Intention

Currently, scholars at home and abroad do not have an unified definition of EI. Although scholars vary widely on EI, there are still similarities in their interpretations of EI. Bird (1988) first proposed the concept of EI and pointed out that EI is a psychological state that makes the thoughts, attention, and behaviors of potential entrepreneurs focus on entrepreneurial activities. As the state of consciousness prior to action process, EI has an imperative role in an individual’s decision to perform new business and is assumed as the best estimator of entrepreneurial-focused behavior (Gartner, 1988; Ajzen, 1991). Thompson (2009) defines EI as a person’s intention to establish innovative businesses and to determinedly seek entrepreneurial opportunities in the future (Shahab et al., 2019). EI has been explicated in the extent literature as the psychological state of the person that stimulates the human desire to launch novel businesses or foster some novel extensions within the present streams of the business. This study has defined EI as a state of psychological preparation for an individual to establish an enterprise based on cognitive experience, emotional factors, and external environmental factors.

### Entrepreneurial Attitude

Social psychology defines attitude as the representation of individual cognition, including the subjective evaluation of oneself, others, affairs, activities, events, and so on. It has an important effect on individual responses and behaviors. EA refers to the level to which an individual is unwilling or inclined to engage in entrepreneurial activity (Ajzen, 1991). The reason why EA is considered as one of the factors affecting EI is that scholars from different backgrounds, cultures, and research samples have proven that EA is the most significant predictor of EI. EA positively and significantly affects EI (Seng Te et al., 2019; Otache et al., 2021; Wijayati et al., 2021). All these studies show that the more positive students’ attitudes toward entrepreneurship, the stronger their intention to start their own business.

The enlisted hypothesis is drawn on the basis of the above-mentioned discussions:

*H1*: EI is positively influenced by EA.

### Subjective Norms

SN is one of the important determinants of the TPB. SN refers to the perception of the person on whether intimate relationships in the social environment, such as colleagues, friends, and family, support a certain behavior, and on the impact of such an evaluation on the individual (Ajzen,1991). Many studies have shown that the influence of SN on intention has high explanatory power and is extremely important for increasing EI. Lai and To (2020) conducted a survey of 220 young Chinese adults and found that SN significantly influences EI. Shi et al. (2020) report that there exists a significantly positive association between SN and EI among university students in Zhejiang Province, China. Other studies collected data from different countries and regions to underpin the association between SN and EI. Their studies have established consistent implications that SN facilitates the formation of EI (Mahmood et al., 2017; Palamida et al., 2018; Tarapuez et al., 2018).

Based on the proposed arguments, we have postulated the below hypothesis:

*H2*: EI is positively influenced by SN.

### Entrepreneurial Self-Efficacy

Bandura (1977), an American psychologist, was the first to propose the idea of self-efficacy by terming it as the judgment and self-evaluation of the person to complete a specific set of behaviors. Bandura’s concept of self-efficacy is used to derive the concept of ESE. Chen et al. (1998) defined ESE as the strength of individuals to believe that they can successfully perform the roles and tasks of entrepreneurs. Individuals with a high sense of ESE tend to have firm beliefs, make more unremitting efforts in the face of tasks, and cope with more difficult challenges. People with low ESE often have to deal with stress and depression, which limit or impair their skill level.

The role of ESE in entrepreneurship research has received increasing attention, especially in research on the factors influencing EI. Self-efficacy is reported as a good predictor of start-up intention (Krueger et al., 2000; Zakaria and Nordin, 2020). The application and practice of social cognition theory (SCT) in entrepreneurship research indicates that EI and success are largely influenced by ESE, while Pelegrini and de Moraes (2021) revealed that the ESE of university students in developing countries significantly positively predicted EI. Meanwhile, gender difference lies in female ESE, which has a greater impact on EI. Chu et al. (2020) conducted a questionnaire survey among 312 Chinese students who participated in early entrepreneurial practice and found that all aspects of ESE had a significantly positive effect on EI. With an exception, Amofah et al. (2020) studied 159 MBA students from two private universities in Ghana and found that ESE had no significant impact on college students’ EI.

Consistent with the above arguments, the below hypothesis is also postulated:

*H3*: EI is positively influenced by ESE.

### Emotional Competency

Goleman (1998) defined EC as a kind of learning ability based on emotional intelligence, and a high-level EC contributes to excellent work performance. Additionally, Goleman recommended that there are two dimensions of EC: social and personal and skills. Personal skills are the ability to manage oneself. Among these personal skills, he identified three main abilities: self-awareness, self-regulation, and motivation. In terms of social skills, he suggested two abilities: empathy and social skills. Pradhan and Nath (2012) found that there is a significant positive correlation between emotional intelligence and entrepreneurial orientation. Subsequently, a large number of researchers began to pay attention to the relationship between EC and EI and conducted investigations. Huezo-Ponce et al. (2020) investigated students in different entrepreneurial ecosystems in Guadalajara, Jalisco, and Mexico and found that EC directly and positively affects EA and ESE but does not directly and positively affect EI. However, EA and ESE as opportunity perceptions had a directly positive impact on the enterprise performance of college students. Similarly, Wu and Tian (2021) conducted a survey of vocational college students in China and came to the same conclusion: EC completely affects EI through the mediators EA and ESE. Therefore, it can be concluded that with the improvement in their cognitive background, students with stronger EC are more inclined to develop the intention of launching a new business. Wen et al. (2020) found a significant positive correlation between ESE and EC. With the improvement in the EC level of vocational college students, ESE will increase. The lower the EC, the faster the improvement in ESE. The higher the EC, the more stable the ESE is. In China, having a high EC is extremely important for coping with the challenges of starting a business (Chu et al., 2020).

We have drawn the enlisted hypotheses on basis of the above arguments:

*H4*: EA is positively influenced by EC.

*H5*: ESE is positively influenced by EC.

*H6*: EI is positively influenced by EC.

### Entrepreneurial Education

EE aims to promote the fundamental quality of entrepreneurship and generate a kind of personality as the goal, not only to encourage the individual’s consciousness of innovation, EE, entrepreneurship, and innovation spirit, but also to face the whole society, for those who intend to pursue creative businesses, entrepreneurship, the entrepreneurial venture group, a phased hierarchical education of innovative training, thinking, and entrepreneurship capacity of practice (Frank et al., 2007; Liñán et al., 2011). Education services and training are majorly significant in emerging market countries, where low levels of business and technical skills can hinder motivated people from launching innovative ventures (Gnyawali and Fogel, 1994). According to self-determination theory, human capital theory, and ESE theory, EE is positively correlated with students’ EI, because EE can provide enough knowledge and skills to motivate students to develop entrepreneurial careers. Gieure et al. (2019) revealed that students are more willing to gain entrepreneurial skills through effective training and education, and those with higher skills are more confident in themselves and more inclined to generate entrepreneurial ideas. In other words, ESE plays an important mediating role in the EE interpretation of EI. Boldureanu et al. (2020) pointed out that in entrepreneurship-focused educational programs, acquaintance with effective entrepreneurial models can be an important factor in fostering students’ confidence in their entrepreneurial capacity and improving their attitude toward entrepreneurship. This finding is consistent with previous studies (Liñán et al., 2011). Jiang et al. (2017) took Chinese students majoring in social science and engineering science as research subjects and found that the quality of EE had a positive impact on EI, among which ESE played a mediating role. Bi and Collins (2021) confirmed the importance of both traits and mindsets in the development of students’ ESE and identified the bridging role of behavioral skills. Mozahem and Adlouni (2021) tested and validated a sample of a total of 560 students from 4 private Lebanon universities and found that entrepreneurship courses led to an increase in ESE in the sample used.

The below hypotheses are presented based on the above arguments:

*H7*: EA is positively influenced by EE.

*H8*: ESE is positively influenced by EE.

*H9*: EI is positively influenced by EE.

**Figure 1:**
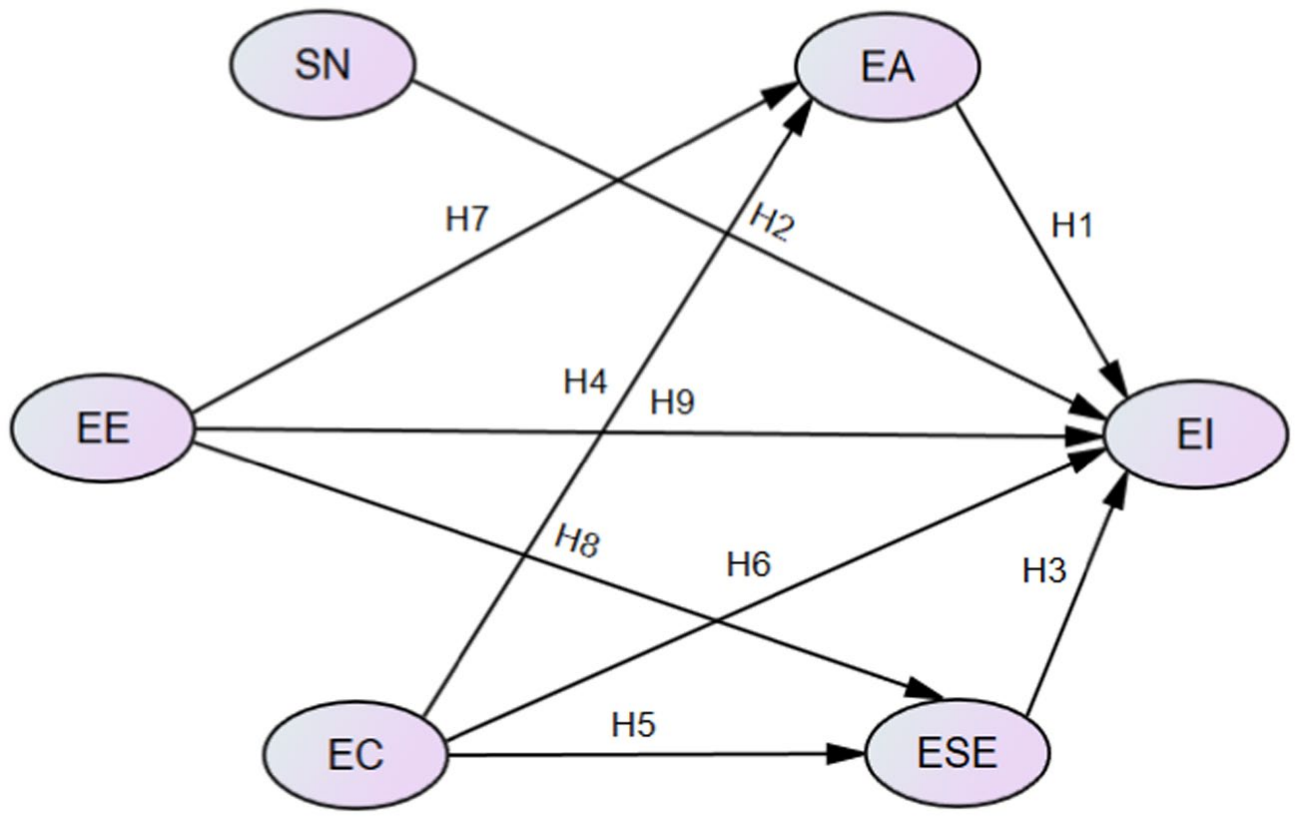
Conceptual framework. SN = subjective norms, EA = entrepreneurial attitude, ESE = entrepreneurial self-efficacy, EI = entrepreneurial intention, EC = emotional competency, EE = entrepreneurial education.

To put it in a nutshell, after taking into account that EI represents the intention to become an entrepreneur or launch a new business, we use an EC perspective and EE theory to analyze the relationships among EC, cognitive factors (EA, SN, ESE), EE, and EI and extend the TPB model. The model to be tested is shown in Figure 1.

## Materials and Methods

### Sample Description

In order to test this research proposition, an empirical study was conducted on students of vocational colleges in Nanchang city, Jiangxi Province, China through a questionnaire survey. Student groups have always been an important object of research on EI (e.g., Kolvereid, 1996; Krueger et al., 2000; Padilla-Angulo, 2017; Bell, 2019; Gieure et al., 2019; Neneh, 2020), and universities are crucial for cultivating students’ motivation and ability to participate in entrepreneurial activities effectively. In addition, previous empirical data have shown that Chinese students have strong entrepreneurial potential and high entrepreneurial awareness (e.g., Elston and Weidinger, 2018; Liu et al., 2019; Shi et al., 2020). Therefore, we believe that studying the EI of college students can provide practical and effective insights into China’s environment.

Questionnaires were distributed and collected on the Internet. The study was ethical and completely confidential and anonymous, with all questions related to the study itself. At the beginning of the questionnaire, the participants were clear about the purpose of the survey and volunteered to participate. Questionnaires that take less than 10 min to complete will be excluded. A total of 382 students completed the questionnaires. The sample was mainly female (68.6%). Most of the students came from rural areas (82.5%).

The demographic information of respondents is shown in Table 1.

**Table 1:** Demographic information of respondents.

Demographic variables		F	%
Gender	Male	120	31.40
	Female	262	68.60
Grade	Freshman	265	69.40
	Sophomore	102	26.70
	Junior	15	3.90
Major field	Science and Technology	136	35.60
	Literature and History	2	0.52
	Economic management	116	30.37
	Others	128	33.51
Origin of student	Urban	67	17.54
	Rural	315	82.46

*N = 382; F: Frequency; %: Percent*.

**Table 2:** Items used to measure research constructs.

SN		
Definition: It refers to an individual’s perception of whether close relationships, such as colleagues, friends, and family, support a certain behavior in the social environment, and the impact of such evaluation on an individual.		
SN1	If I decided to create a firm, my closest family would approve of that decision.	Kolvereid (1996) and Liñán and Chen (2009)
SN2	If I decided to create a firm, my closest friends would approve of that decision.	
SN3	If I decided to create a firm, people who are important to me would approve of that decision.	
**EA**		
Definition: The degree to which an individual is unwilling or inclined to engage in entrepreneurial activity.		
EA1	Being an entrepreneur implies more advantages than disadvantages to me.	Liñán and Chen (2009)
EA2	A career as entrepreneur is attractive for me.	
EA3	If I had the opportunity and resources, I’d like to start a firm.	
EA4	Being an entrepreneur would entail great satisfactions for me.	
EA5	Among various options, I would rather be an entrepreneur.	
**EE**		
Definition: It is a kind of education and training service aiming at improving the basic quality of entrepreneurship and producing a kind of personality.		
EE1	The university promotes the students’ capability required for entrepreneurship.	Denanyoh et al. (2015)
EE2	The university enhances students’ skills related to entrepreneurship.	
EE3	The university gives students with applicable information and assist students on how to initiate a venture.	
EE4	I believe that entrepreneurship matters can be initiated through education.	
**EC**		
Definition: It is a kind of learning ability based on emotional intelligence, and having good EC contributes to excellent work performance.		
EC1	I am able to recognize my own emotions and their effect on my actions.	Fernández-Pérez et al. (2019)
EC2	I consider myself a person who is flexible and capable of addressing changes.	
EC3	I like to push myself to improve or to meet a certain criterion of excellence.	
EC4	I am able to understand the feelings and viewpoints of others and I am actively interested in the things they care about.	
EC5	I have the ability to negotiate and resolve disagreements.	
**ESE**		
Definition: It is the strength of individuals to believe that they can successfully perform the roles and tasks of entrepreneurs.		
ESE1	I can work productively under continuous stress, pressure, and conflict.	Tsai et al. (2014) and Liñán (2008)
ESE2	I can originate new ideas and products.	
ESE3	I can develop and maintain favorable relationships with potential investors.	
ESE4	I can see new market opportunities for new products and services.	
ESE5	I can recruit and train key employees.	
ESE6	I can develop a working environment that encourages people to try out something new.	
**EI**		
Definition: A state of psychological preparation established by individuals based on cognitive experience, emotional factors, and external environmental factors.		
EI1	My professional goal is becoming an entrepreneur.	Liñán and Chen (2009)
EI2	I will make every effort to start and run my own firm.	
EI3	I am determined to create a firm in the future.	
EI4	I have very seriously thought of starting a firm.	
EI5	I have got the firm intention to start a firm someday.	

*SN = subjective norms, EA = entrepreneurial attitude, ESE = entrepreneurial self-efficacy, EI = entrepreneurial intention, EC = emotional competency, EE = entrepreneurial education*.

### Research Instrument

As the research model (Figure 1) is presented by the authors, none of the available tools are appropriate. We integrated and appropriately modified the tools previously validated in other environments to form the questionnaire for this study. In the actual test, the questions were translated into the Chinese language so that the participants could understand the research questions more accurately. Therefore, a 5-point Likert scale ranging from 5 (strongly agreed) to 1 (strongly disagreed) was used to measure all relevant items. The variables were adopted as follows: For the measurement of SN, the three questions were drawn from the scales of Kolvereid (1996) and Liñán and Chen (2009). For the measurement of EA, five questions were drawn from the scale of Liñán and Chen (2009). For the measurement of EE, four questions were drawn from the scale of Denanyoh et al. (2015). For the measurement of EC, five questions were drawn from the scale of Fernández-Pérez et al. (2017). For the measurement of ESE, six questions were drawn from the scales of Tsai et al. (2016) and Liñán (2008). For the measurement of EI, five questions were drawn from the scale of Liñán and Chen (2009). All items used in this study list in Table 2.

### Data Analysis

As suggested by Anderson and Gerbing (1988), we adopted a two-step approach to the structural equation model (SEM). First, the measurement model was verified by conducting a confirmatory factor analysis (CFA). SEM was used to test the relationship between the structures. Finally, according to Hair et al. (2006), we applied fit indices from different categories to test model fitting: absolute fit indices, which evaluate the efficiency at which the proposed model regenerates the observed data, indices of parsimony, which is the same as the absolute fit indices except that it considers the complexity of the model, and incremental fit indices, which examine how effectively a specified model fits comparatively to the alternative-baseline model. AMOS 24.0 was used to undertake all analyses.

## Results

### Descriptive Statistics

Table 3 represents the descriptive statistics of the constructs.

**Table 3:** Descriptive statistics.

	Mean	SD	Skewness	Kurtosis
SN	3.586	0.762	−0.439	0.946
EE	3.798	0.711	−0.965	2.253
EC	3.698	0.756	−0.813	1.336
EA	3.678	0.779	−0.504	0.351
ESE	3.562	0.747	−0.355	0.291
EI	3.559	0.766	−0.218	−0.066

*SN = subjective norms, EA = entrepreneurial attitude, ESE = entrepreneurial self-efficacy, EI = entrepreneurial intention, EC = emotional competency, EE = entrepreneurial education*.

Prior to the CFA, the measurement model was assessed for construct validity and reliability. The descriptive statistics showed that all mean values are greater than the midpoint of 3.50, varying from 3.559 to 3.798. Additionally, the standard deviation (S.D) ranged from 0.711 to 0.779. The skew index ranged between −0.965 and − 0.218 while the kurtosis index varied from −0.066 to 1.336. Both Skew and Kurtosis indices are acceptable and reflective of univariate normality. Thus, the data gathered for this study is considered sufficient for structural equation modeling.

### Test of the Measurement Model

The purpose of the CFA test is to determine if the association between a factor and its corresponding measure fulfills the theoretical associations presented by researchers. The assessment of significance is used to establish the model fit through the t-values of the individual item loadings. This study has also used the root mean square error of approximation (RMSEA), *x*^2^ statistics, standardized root mean residual (SRMR), Tucker–Lewis index (TLI), and comparative fit index (CFI). The chi-square divided by degrees of freedom (*x*^2^/df) should not be greater than 3 (Carmines, 1981) and the CFI and TLI should both surpass 0.90 (Preacher and Hayes, 2008) for a model to be considered a good fit. Both SRMR and RMSEA should not be greater than 0.08 in order to be considered sufficient (Hair et al., 2006). Additionally, as per the suggestion of Hair et al. (2021), the average variance extracted (AVE) value needs to exceed 0.5, as the composite reliability (CR) value should be greater than 0.8.

The measurement model also exhibits an adequate model fit (*x*^2^ = 791.320, *x*^2^/df = 2.362, GFI = 0.868, AGFI = 0.839, TLI = 0.942, CFI = 0.949, RMSEA = 0.060, SRMR = 0.042). In sum, our test results indicate the appropriateness of the measurement model, which is a reliable indicator of the hypothesized constructs, thus allowing tests of the structural relationships in the various models to proceed.

### Convergent and Discriminant Validities

Convergent validity is to evaluate whether the respective indicators are measuring the construct they want to measure. Table 4 demonstrates the factor loading of each item on the constructs in the measurement model. All parameter estimates are significant at a *p* 0.05 level. The AVE values ranged between 0.563 for EC and 0.720 for EE, which are above the threshold value of 0.50. Cronbach’s alpha is used to calculate the internal consistency of all constructs. As shown in Table 4, Cronbach’s Alpha values were considered high in the range of 0.834–0.949 (Nunnally and Bernstein, 1994). Therefore, convergence validity was established for all measurement items in this study.

**Table 4:** Convergence validity.

			Unstd.	S.E.	*t*-value	*p*	std.	SMC	CR	AVE	Cronbach’s alpha
SN1	<−--	SN	1.000				0.873	0.561	0.910	0.771	0.850
SN2	<−--	SN	1.004	0.067	14.976	***	0.858	0.674			
SN3	<−--	SN	1.071	0.071	15.112	***	0.903	0.745			
EA1	<−--	EA	1.000				0.835	0.496	0.924	0.709	0.948
EA2	<−--	EA	1.143	0.080	14.217	***	0.794	0.612			
EA3	<−--	EA	1.066	0.073	14.655	***	0.878	0.653			
EA4	<−--	EA	1.105	0.074	15.009	***	0.825	0.689			
EA5	<−--	EA	1.256	0.081	15.481	***	0.875	0.740			
EC1	<−--	EC	1.000				0.770	0.354	0.904	0.654	0.834
EC2	<−--	EC	1.515	0.138	10.967	***	0.780	0.608			
EC3	<−--	EC	1.278	0.121	10.569	***	0.850	0.533			
EC4	<−--	EC	1.233	0.121	10.182	***	0.810	0.473			
EC5	<−--	EC	1.367	0.128	10.683	***	0.830	0.552			
EI1	<−--	EI	1.000				0.912	0.790	0.960	0.799	0.896
EI2	<−--	EI	0.848	0.040	21.379	***	0.921	0.661			
EI3	<−--	EI	1.015	0.035	28.673	***	0.921	0.867			
EI4	<−--	EI	1.008	0.041	24.315	***	0.887	0.750			
EI5	<−--	EI	1.060	0.039	26.930	***	0.906	0.821			
EE1	<−--	EE	1.000				0.891	0.753	0.961	0.831	0.919
EE2	<−--	EE	1.040	0.036	28.985	***	0.952	0.912			
EE3	<−--	EE	1.085	0.037	29.092	***	0.955	0.916			
EE4	<−--	EE	0.953	0.044	21.787	***	0.910	0.696			
ESE1	<−--	ESE	1.000				0.783	0.552	0.938	0.715	0.949
ESE2	<−--	ESE	1.131	0.069	16.480	***	0.831	0.691			
ESE3	<−--	ESE	0.999	0.061	16.481	***	0.939	0.691			
ESE4	<−--	ESE	1.058	0.065	16.342	***	0.875	0.681			
ESE5	<−--	ESE	1.011	0.060	16.721	***	0.847	0.709			
ESE6	<−--	ESE	0.897	0.057	15.703	***	0.795	0.632			

Unstd. = unstandardized factor loading; std. = standardized factor loading; S.E. (standard error) is an estimate of the standard error of the covariance; CR is the critical ratio obtained by dividing the estimate of the covariance by its standard error; AVE: average variance extract; SMC = the square of the normalization coefficient; *** denotes p < 0.001, SN = subjective norms, EA = entrepreneurial attitude, ESE = entrepreneurial self-efficacy, EI = entrepreneurial intention, EC = emotional competency, EE = entrepreneurial education.

Discriminant validity denotes the significant difference or low correlation between the potential variables represented by one or another facet. To examine the discriminant validity between the constructs, the criteria proposed by Fornell and Larcker (1981) were adapted in this study. The square root of the AVE (diagonal) of the measurement model is greater than the correlation coefficient (off-diagonal) between the latent variable and other latent variables in Table 5 below. Then, it indicates good discriminant validity.

**Table 5:** Discriminant validity.

	EA	EC	EE	EI	ESE	SN
EA	**0.842**					
EC	0.622	**0.809**				
EE	0.486	0.391	**0.912**			
EI	0.745	0.52	0.603	**0.894**		
ESE	0.788	0.68	0.522	0.788	**0.846**	
SN	0.572	0.586	0.335	0.476	0.551	**0.878**

*SN = subjective norms, EA = entrepreneurial attitude, ESE = entrepreneurial self-efficacy, EI = entrepreneurial intention, EC = emotional competency, EE = entrepreneurial education*. The bold figure of the diagonal is the square root of average variance extracted (AVE).

### Path Coefficient Analysis

The major purpose of the path coefficient is to report the extent and impact significance of the independent variables related to the dependent variable. As indicated by Hair et al. (2021), the authors in this study have put forward a directional hypothesis for H1 to H9, revealing that the value of p must be below 0.05 and the t-value should be greater than 1.96. Consistent with Table 5, hypothese2 was rejected because the criteria were not fulfilled.

The study findings indicate that EA significantly impact the EI (*β* = 0.426, *p* < 0.001), ESE (*β* = 0.79, *p* < 0.001), and EE (*β* = 0.113, *p* < 0.05), as a result supporting Hypotheses 1, 3, and 9. Although, SN (*β* = 0.02, *p* > 0.05) has no significant influence on EI. Furthermore, EC had a significant effect on EI (*β* = −0.435, *p* < 0.001), EA (*β* = 0.749, *p* < 0.001), and ESE (*β* = 0.776, *p* < 0.001). EE also had a significantly positive impact on EA (*β* = 0.16, *p* < 0.001), and ESE (*β* = 0.189, *p* > 0.05). These results provide support for hypotheses 4, 5, 6, 7, and 8. In total, eight of the nine hypotheses are supported.

### Mediating Testing

The Bootstrap method currently serves as the ideal test method for mediating effects (Preacher and Hayes, 2008). A significant mediating effect is considered in cases where the confidence interval of the computed indirect effect does not contain zero.

Tables 6 and 7 show the output of this test, which reveal that EI is affected by EE through EA and ESE, indicating that EA and ESE are mediators. EE directly and significantly affects EI, and EE partially affects EI through EA and ESE. Meanwhile, EC directly and significantly affects EI. EC partially affects EI through EA and ESE.

**Table 6:** Summary of the hypothesis tests.

Hypotheses	Path	Path coefficient (*β*)	*t*-value	*p*-value	Results
H1	EA → EI	0.426	5.215	***	Accepted
H2	SN → EI	0.02	0.346	ns	Rejected
H3	ESE → EI	0.79	7.46	***	Accepted
H4	EC → EA	0.749	9.322	***	Accepted
H5	EC → ESE	0.776	9.862	***	Accepted
H6	EC → EI	−0.435	−2.967	**	Accepted
H7	EE → EA	0.16	3.612	***	Accepted
H8	EE → ESE	0.189	4.611	***	Accepted
H9	EE → EI	0.113	2.541	*	Accepted

*** denotes p < 0.001, ** denotes p < 0.01, * denotes p < 0.5,,ns = non-significant; SN = subjective norms, EA = entrepreneurial attitude, ESE = entrepreneurial self-efficacy, EI = entrepreneurial intention, EC = emotional competency, EE = entrepreneurial education.

**Table 7:** The mediating testing result.

Parameter	Path coefficient (*β*)	SE	Bias-corrected 95%CI			Percentile 95%CI			Results
			Lower	Upper	*p*-value	Lower	Upper	*p*-value	
**Indirect effects**									
EE → EA → EI	0.068	0.04	0.005	0.16	0.037	0.007	0.149	0.067	Accepted
EE → ESE → EI	0.15	0.051	0.053	0.256	0.003	0.048	0.25	0.004	Accepted
EC → EA → EI	0.319	0.098	0.15	0.534	0.001	0.154	0.543	0.001	Accepted
EC → ESE → EI	0.613	0.13	0.413	0.93	0	0.41	0.922	0	Accepted
**Direct effects**									
EE → EI	0.122	0.098	0.04	0.351	0.048	0.06	0.324	0.027	Accepted
EC → EI	−0.731	0.348	−1.624	−0.255	0.002	−1.541	−0.231	0.002	Accepted
**Total effects**									
EE → EI	0.358	0.117	0.128	0.591	0.001	0.117	0.584	0.002	Accepted
EC → EI	0.834	0.219	0.440	1.303	0.004	0.219	0.510	0.002	Accepted

*SN = subjective norms, EA = entrepreneurial attitude, ESE = entrepreneurial self-efficacy, EI = entrepreneurial intention, EC = emotional competency, EE = entrepreneurial education*.

## Discussion and Implications

### Discussion

This study used TPB, SCT, and EE to investigate the influence of EA, SN, ESE, EC, and EE on EI. As mentioned above, TPB is apparently inadequate to explain EI, because it only considers cognitive factors. Therefore, ESE, EC, and EE were introduced through SCT and other literature reviews. By integrating the above factors, we were in a position to move beyond the explanations presented in the TPB framework, which consequently improved the explanatory power of the research model proposed in this study.

As expected, the results of this study showed that EA and ESE were the two most significant factors affecting EI. As predicted, our study with Chinese vocational college students as the research sample proved that EA and ESE have a positive influence on EI, which is parallel to research findings (Law and Breznik, 2016; Cavazos-Arroyo et al., 2017). It is entirely possible that a person who has a positive attitude toward starting a business, or a person who is confident in their capacity to establish a new business and has the courage to face the follow-up challenges, is more prone to start a business.

Surprisingly, our study found that the SN was not a significant predictor of EI. This is inconsistent with some previous studies (Ajzen, 1991; Cavazos-Arroyo et al., 2017; Shi et al., 2020). Our results suggest that important people, such as family and friends, have little bearing on whether an individual chooses to start a business. This means that college students may already have a relatively independent idea of their future employment choices. The research of Doanh and Bernat (2019) and Hongdiyanto et al. (2020) has confirmed our findings and held that SN is not an important factor in explaining EI, further pointing out that SN affects EI through EA and ESE.

EE had a significant and positive influence on EI. The results of the mediation test show that EA and ESE play partial mediating roles in EE and EI, which is consistent with previous research (Jiang et al., 2017; Boldureanu et al., 2020). The skills and knowledge needed to launch a business can be acquired through EE. Therefore, by teaching students the knowledge and skills needed for future entrepreneurship, it can both directly promote individuals to produce EI and promote EI by producing more positive EA or higher ESE. Sun et al. (2017) did an in-depth study on this, which not only supplemented the TPB model but also pointed out what specific elements of EE influence EA and ESE.

EC partially mediates EI through EA and ESE. This fact confirms previous studies by other authors (Fernández-Pérez et al., 2017; Wen et al., 2020). In other words, the improvement in EC will provide value for the precognitive factors (EA, ESE) of EI. When college students’ EC improve, they feel more convinced about their entrepreneurial capabilities, which allows them to capitalize on the opportunities. Similarly, the more EC students were, the more positive they felt about entrepreneurship and the more likely they were to pursue entrepreneurship as a career choice.

### Theoretical Implications

The findings of this study make several contributions to this field. First of all, this study investigated the factors influencing the EI of vocational college students from different grades and disciplines in the context of COVID-19, which has not been sufficiently studied so far. Secondly, a insignificant relationship between SN and EI was confirmed, revealing the insignificant role of SN in students’ EI in the COVID-19 pandemic. Thirdly, EE partially affects EI through EA and ESE. Meanwhile, EC partially affects EI through EA and ESE.

### Practical Implications

First, higher vocational colleges should strengthen their innovative education. EE has a significantly positive impact on entrepreneurial consciousness. On the one hand, higher vocational colleges should update their educational ideas according to the background of The Times, integrate the idea of innovation and entrepreneurship into every link between education and teaching, pay attention to improving the comprehensive quality of students, and train students to take the concept of lifelong entrepreneurship as their goal. On the other hand, innovation and EE should attach importance to the status and role of teachers in education and strive to create a team of innovative teachers with excellent quality, solid expertise, and strong practical ability.

Second, the quality of students at higher vocational colleges should be improved. Students should take the initiative to overcome passive thinking, overcome the thought of not being enterprising and not seeking to make progress, and instead, have an objective and scientific understanding of their own strengths and weaknesses, and be full of confidence and determination in their entrepreneurial dreams and prospects. Focus on learning, cultivating entrepreneurial awareness, and identifying potential entrepreneurial opportunities.

Finally, a good entrepreneurial environment in society should be created (Wu and Wang, 2018). On the one hand, based on the national conditions of the country, the government can support college students’ starting their own businesses by reducing the cost of taxation, discount loans, simplifying the examination and approval process, etc., and can also set up entrepreneurship guidance institutions to give timely and effective guidance to inexperienced students. On the other hand, social media is used to change the social value orientation and gradually improve people’s inherent impression of entrepreneurship so that students can get support from their families and friends.

### Research Limitations and Future Works

As with all empirical studies, the current study is not free of limitations. First, the study sample included only 382 students from technical and vocational colleges in Jiangxi Province of China. Therefore, the research sample should be wider than this paper and further research should also include student subjects from other provinces or countries to discover potential differences in the influencing factors of students’ EI. Second, because the aim of this research study is not to assess the business practices themselves, it fails to highlight how EI translates into actions over time. Therefore, further longitudinal studies are needed to examine this issue and validate these results in different entrepreneurial settings. Third, underclassmen and upperclassmen seem to be important factor in EE because of their study experience in vocational colleges. These analysis results might be useful in conducting the effectiveness of EE. Therefore, it is worth supplementing in subsequent studies. Finally, the relationship between EI and individual EC is also worth exploring considering that EE can be received in active and passive ways.

## Data Availability Statement

The raw data supporting the conclusions of this article will be made available by the authors, without undue reservation.

## Ethics Statement

The studies involving human participants were reviewed and approved by the Ethics Committee of Psychology and Behavioral Sciences, Tianjin University. Written informed consent for participation was not required for this study in accordance with the national legislation and the institutional requirements.

## Author Contributions

XW and ZO: conceptualization. XF, YT, and XN: data curation. XF, YT, and TY: writing original draft. TY, XX, YW, and QH: writing—review and editing. All authors contributed to the article and approved the submitted version.

## Funding

This research was supported by the key base project of Humanities and Social Sciences in Colleges and Universities of Jiangxi Province (JD21069); Science and Technology Project of Education Department of Jiangxi Province in 2018 (No: GJJ190590); Jiangxi Social Science Planning Project in 2018 (No: 18JY21); Humanities and Social Science Project of Universities in Jiangxi Province in 2020 (No: JY20219); and Doctoral Research Foundation of Jiangxi Science and Technology Normal University in 2021 (No: 2021BSQD20).

## Conflict of Interest

The authors declare that the research was conducted in the absence of any commercial or financial relationships that could be construed as a potential conflict of interest.

## Publisher’s Note

All claims expressed in this article are solely those of the authors and do not necessarily represent those of their affiliated organizations, or those of the publisher, the editors and the reviewers. Any product that may be evaluated in this article, or claim that may be made by its manufacturer, is not guaranteed or endorsed by the publisher.

## References

[ref1] Ajzen I. (1985). “From intentions to actions: a theory of planned behavior,” in action Control. SSSP Springer Series in Social Psychology. (eds) Kuhl J. Beckmann J. (Berlin, Heidelberg: Springer). doi: 10.1007/978-3-642-69746-3_2

[ref2] Ajzen I. (1991). The theory of planned behavior. Organ. Behav. Hum. Decis. Process. 50, 179–211. doi: 10.1016/0749-5978(91)90020-T

[ref3] Amofah K. Saladrigues R. Akwaa-Sekyi E. K. (2020). Entrepreneurial intentions among MBA students. Cogent Busi. Manag 7:1832401. doi: 10.1080/23311975.2020.1832401

[ref4] Anderson J. C. Gerbing D. W. (1988). Structural equation modeling in practice: a review and recommended two-step approach. Psychol. Bull. 103, 411–423. doi: 10.1037/0033-2909.103.3.411

[ref5] Armitage C. J. Conner M. (2001). Efficacy of the theory of planned behaviour: a meta-analytic review. Br. J. Soc. Psychol. 40, 471–499. doi: 10.1348/014466601164939, PMID: 11795063

[ref6] Bae T. J. Qian S. Miao C. Fiet J. O. (2014). The relationship Between entrepreneurship education and entrepreneurial intentions: a meta-analytic review. Entrep. Theory Pract. 38, 217–254. doi: 10.1111/etap.12095

[ref01] Bandura A. (1977). Self-efficacy: toward a unifying theory of behavioral change. Psychol. Rev. 84:191. 847061 10.1037//0033-295x.84.2.191

[ref7] Bell R. (2019). Predicting entrepreneurial intention across the university. Education + Training 61, 815–831. doi: 10.1108/et-05-2018-0117

[ref8] Bi Q. C. Collins J. (2021). Proactivity, mindsets and the development of students’ entrepreneurial self-efficacy: behavioural skills as the catalyst. Journal of the Royal Society of New Zealand, 51, 1–13. doi: 10.1080/03036758.2021.1999993 PMC11485731 39440190

[ref9] Bird B. (1988). Implementing entrepreneurial ideas: the case for intention. Acad. Manag. Rev. 13, 442–453. doi: 10.5465/amr.1988.4306970

[ref10] Boldureanu G. Ionescu A. M. Bercu A. M. Bedrule-Grigoruta M. V. Boldureanu D. (2020). Entrepreneurship education through successful entrepreneurial models in higher education institutions. Sustainability 12:1267.

[ref11] Carmines E. G. (1981). Analyzing models with unobserved variables. Social measurement: current issues, 80.

[ref12] Cavazos-Arroyo J. Puente-Díaz R. Agarwal N. (2017). An examination of certain antecedents of social entrepreneurial intentions among Mexico residentes. Rev. Busi. Manag. 19, 180–218. doi: 10.7819/rbgn.v19i64.3129

[ref13] Chen C. C. Greene P. G. Crick A. (1998). Does entrepreneurial self-efficacy distinguish entrepreneurs from managers? J. Bus. Ventur. 13, 295–316. doi: 10.1016/S0883-9026(97)00029-3

[ref14] Chu C. C. Bin S. Yang H. L. Zheng M. Q. Li B. B. (2020). Emotional competence, entrepreneurial self-efficacy, and entrepreneurial intention: a study based on China college Students’ social entrepreneurship project. Front. Psychol. 11:547627. doi: 10.3389/fpsyg.2020.547627, PMID: 33312146 PMC7704431

[ref15] Denanyoh R. Adjei K. Nyemekye G. E. (2015). Factors that impact on entrepreneurial intention of tertiary students in Ghana. Int. J. Busi. Soc. Res 5, 19–29. doi: 10.18533/ijbsr.v5i3.693

[ref16] Doanh D. C. Bernat T. (2019). Entrepreneurial self-efficacy and intention among vietnamese students: a meta-analytic path analysis based on the theory of planned behavior. Procedia Computer Sci 159, 2447–2460. doi: 10.1016/j.procs.2019.09.420

[ref17] Elston J. A. Weidinger A. (2018). Entrepreneurial intention and regional internationalization in China. Small Bus. Econ. 53, 1001–1015. doi: 10.1007/s11187-018-0114-5

[ref18] Fernández-Pérez V. Montes-Merino A. Rodríguez-Ariza L. Galicia P. E. A. (2017). Emotional competencies and cognitive antecedents in shaping student’s entrepreneurial intention: the moderating role of entrepreneurship education. Int. Entrep. Manag. J. 15, 281–305. doi: 10.1007/s11365-017-0438-7

[ref001] Fernández-Pérez V. Montes Merino A. Rodríguez-Ariza L. Alonso-Galicia P. (2019). Emotional competencies and cognitive antecedents in shaping student’s entrepreneurial intention: the moderating role of entrepreneurship education. International Entrepreneurship and Management Journal 15. doi: 10.1007/s11365-017-0438-7

[ref19] Fornell C. Larcker D. F. (1981). Evaluating structural equation models with unobservable variables and measurement error. J. Mark. Res. 18, 39–50. doi: 10.1177/002224378101800104

[ref20] Frank H. Lueger M. Korunka C. (2007). The significance of personality in business start-up intentions, start-up realization and business success. Entrepreneurship Reg. Devel 19, 227–251. doi: 10.1080/08985620701218387

[ref21] Frye C. C. (2018). Accelerating physician entrepreneurship: perspective of a recently graduated medical student. Medical Innovation, 179–190. doi: 10.1016/b978-0-12-814926-3.00018-8

[ref22] Gartner W. B. (1988). “Who is an entrepreneur?” is the wrong question. Am. J. Small Busi. 12, 11–32. doi: 10.1177/104225878801200401

[ref23] Gieure C. Benavides-Espinosa M. del M. Roig-Dobón S. (2019). Entrepreneurial intentions in an international university environment. Int. J. Entrep. Behav. Res. 25, 1605–1620. doi: 10.1108/ijebr-12-2018-0810

[ref24] Gnyawali D. R. Fogel D. S. (1994). Environments for entrepreneurship development: key dimensions and research implications. Entrep. Theory Pract. 18, 43–62. doi: 10.1177/104225879401800403

[ref25] Goleman D. (1998). Working with Emotional Intelligence. New York: Bantam Books.

[ref26] Hair J. F. Black W. C. Babin B. J. Anderson R. E. Tatham R. L. (2006). Multivariate data analysis Pearson Prentice Hall. humans: critique and reformulation. J. Abnorm. Psychol. 6th Edn. 87, 49–74.

[ref27] Hair J. F. Jr. Hult G. T. M. Ringle C. M. Sarstedt M. (2021). A primer on partial least squares structural equation modeling (PLS-SEM). London: Sage Publications.

[ref28] Hongdiyanto C. Teofilus T. Sutrisno T. Dewanti P. (2020). The effect of entrepreneurial learning towards entrepreneurial intention of indonesian women. J. Asian Fin. Eco. Busi 7, 573–582. doi: 10.13106/jafeb.2020.vol7.no9.573

[ref29] Huezo-Ponce L. Fernández-Pérez V. Rodríguez-Ariza L. (2020). Emotional competencies and entrepreneurship: modeling universities. Int. Entrep. Manag. J. 17, 1497–1519. doi: 10.1007/s11365-020-00683-w

[ref31] Jiang H. Xiong W. Cao Y. H. (2017). Research on the mechanism of entrepreneurial education quality, entrepreneurial self-efficacy and entrepreneurial intention in social sciences, engineering and science education. Eurasia J. Mathematics Sci. Technol. Educ 13, 3709–3721. doi: 10.12973/eurasia.2017.00754a

[ref32] Kolvereid L. (1996). Prediction of employment status choice intentions. Entrep. Theory Pract. 21, 47–58. doi: 10.1177/104225879602100104

[ref33] Krueger N. F. (2000). The cognitive infrastructure of opportunity emergence. Entrep. Theory Pract. 24, 5–24. doi: 10.1177/104225870002400301

[ref34] Lai L. S. L. To W. M. (2020). E-entrepreneurial intention among young Chinese adults. Asian J. Technol. Innov. 28, 119–137. doi: 10.1080/19761597.2020.1713832

[ref35] Law K. M. Y. Breznik K. (2016). Impacts of innovativeness and attitude on entrepreneurial intention: among engineering and non-engineering students. Int. J. Technol. Des. Educ. 27, 683–700. doi: 10.1007/s10798-016-9373-0

[ref36] Liñán F. (2008). Skill and value perceptions: how do they affect entrepreneurial intentions? Int. Entrep. Manag. J. 4, 257–272. doi: 10.1007/s11365-008-0093-0

[ref37] Liñán F. Chen Y.-W. (2009). Development and cross-cultural application of a specific instrument to measure entrepreneurial intentions. Entrep. Theory Pract. 33, 593–617. doi: 10.1111/j.1540-6520.2009.00318.x

[ref38] Liñán F. Rodríguez-Cohard J. C. Rueda-Cantuche J. M. (2010). Factors affecting entrepreneurial intention levels: a role for education. Int. Entrep. Manag. J. 7, 195–218. doi: 10.1007/s11365-010-0154-z

[ref02] Liñán F. Rodríguez-Cohard J. C. Rueda-Cantuche J. M. (2011). Factors affecting entrepreneurial intention levels: a role for education. Entrepreneurship Manag. J. 7, 195–218.

[ref39] Liu X. Lin C. Zhao G. Zhao D. (2019). Research on the effects of entrepreneurial education and entrepreneurial self-efficacy on college students’ entrepreneurial intention. Front. Psychol. 10:869. doi: 10.3389/fpsyg.2019.00869, PMID: 31068862 PMC6491517

[ref40] Mahmood T. M. A. T. Mamun A. A. Ibrahim M. D. (2017). Determinants of pre-start-up behavior among rural asnaf millennials. Advanced Science Letters. 24, 8324–8327.

[ref41] Mozahem N. A. Adlouni R. O. (2021). Using entrepreneurial self-efficacy as an indirect measure of entrepreneurial education. Int. J. Manag. Educ 19:100385. doi: 10.1016/j.ijme.2020.100385

[ref42] Neneh B. N. (2020). Entrepreneurial passion and entrepreneurial intention: the role of social support and entrepreneurial self-efficacy. Stud. High. Educ. 47, 587–603. doi: 10.1080/03075079.2020.1770716

[ref43] Nunnally J. C. Bernstein I. H. (1994). Psychometric Theory. New York, NY: McGrawHill.

[ref44] Otache I. Umar K. Audu Y. Onalo U. (2021). The effects of entrepreneurship education on students’ entrepreneurial intentions a longitudinal approach. Educ. Train. 63, 967–991. doi: 10.1108/et-01-2019-0005

[ref45] Padilla-Angulo L. (2017). Student associations and entrepreneurial intentions. Stud. High. Educ. 44, 45–58. doi: 10.1080/03075079.2017.1336215

[ref46] Palamida E. Papagiannidis S. Xanthopoulou D. (2018). Linking young individuals’ capital to investment intentions: comparing two cultural backgrounds. Eur. Manag. J. 36, 392–407. doi: 10.1016/j.emj.2017.06.004

[ref47] Pelegrini G. C. de Moraes G. (2021). Does gender matter? A university ecosystem, self-efficacy and entrepreneurial intention analysis in Brazilian universities. Gender in Management. 37, 271–286. doi: 10.1108/gm-01-2021-0007

[ref48] Pradhan R. K. Nath P. (2012). Perception of entrepreneurial orientation and emotional intelligence. Glob. Bus. Rev. 13, 89–108. doi: 10.1177/097215091101300106

[ref49] Preacher K. J. Hayes A. F. (2008). Asymptotic and resampling strategies for assessing and comparing indirect effects in multiple mediator models. Behav. Res. Methods 40, 879–891. doi: 10.3758/brm.40.3.879, PMID: 18697684

[ref03] Seng Te T. Abdullah A. Rashid Mat A. (2019). Relationship between the selected factors with entrepreneurial career aspirations among students of community colleges in Malaysia. KnE Social Sciences 3, 53–64. doi: 10.18502/kss.v3i12.4073, PMID: 18697684

[ref50] Shahab Y. Ye C. G. Arbizu A. D. Haider M. J. (2019). Entrepreneurial self-efficacy and intention: do entrepreneurial creativity and education matter? Int. J. Entrep. Behav. Res. 25, 259–280. doi: 10.1108/ijebr-12-2017-0522

[ref51] Shane S. Venkataraman S. (2000). The promise of Enterpreneurship as a field of research. Acad. Manag. Rev. 25:217. doi: 10.2307/259271

[ref52] Shi Y. Yuan T. Bell R. Wang J. (2020). Investigating the relationship between creativity and entrepreneurial intention: the moderating role of creativity in the theory of planned behavior. Front. Psychol. 11:1209. 32581972 10.3389/fpsyg.2020.01209 PMC7296068

[ref53] Sun H. Lo C. T. Liang B. Wong Y. L. B. (2017). The impact of entrepreneurial education on entrepreneurial intention of engineering students in Hong Kong. Manag. Decis. 55, 1371–1393. doi: 10.1108/md-06-2016-0392

[ref54] Tarapuez E. Guzmán B. Parra R. (2018). Factores que determinan la intención emprendedora en América Latina. [Determinants of entrepreneurial intention in Latin America]. Suma de Negocios 9, 56–67. doi: 10.14349/sumneg/2018.v9.n19.a7

[ref56] Teo T. Lee C. B. Chai C. S. Wong S. L. (2009). Assessing the intention to use technology among pre-service teachers in Singapore and Malaysia: a multigroup invariance analysis of the technology acceptance model (TAM). Comput. Educ. 53, 1000–1009. doi: 10.1016/j.compedu.2009.05.017

[ref57] Thompson E. R. (2009). Individual entrepreneurial intent: construct clarification and development of an internationally reliable metric. Enterp. Theory Pract. 33, 669–694. doi: 10.1111/j.1540-6520.2009.00321.x

[ref002] Tsai C. L. Chaichanasakul A. Zhao R. Flores L. Y. Lopez S. J. (2014). Development and validation of the strengths self-efficacy scale (SSES). Journal of Career Assessment 22, 221–232.

[ref58] Tsai K. H. Chang H. C. Peng C. Y. (2016). Extending the link between entrepreneurial self-efficacy and intention: a moderated mediation model. Int. Entrep. Manag. J. 12, 445–463. doi: 10.1007/s11365-014-0351-2

[ref59] Wen Y. Chen H. Pang L. Gu X. (2020). The relationship between emotional intelligence and entrepreneurial self-efficacy of Chinese vocational college students. Int. J. Environ. Res. Public Health 17:4511. doi: 10.3390/ijerph17124511, PMID: 32585938 PMC7345360

[ref60] Wijayati D. T. Fazlurrahman H. Hadi H. K. Arifah I. D. C. (2021). The effect of entrepreneurship education on entrepreneurial intention through planned behavioural control, subjective norm, and entrepreneurial attitude. J. Glob. Entrep. Res. 11, 1–14. doi: 10.1007/s40497-021-00298-7

[ref04] Wu X. Tian Y. (2021). Predictors of entrepreneurship intention among students in vocational colleges: a structural equation modeling approach. Front. Psychol. 12:797790. 35095683 10.3389/fpsyg.2021.797790 PMC8790017

[ref61] Wu X. Wang M. (2018). Selection of cooperative enterprises in vocational education based on ANP. Educ. Sci. Theory Prac 18, 1507–1515. doi: 10.12738/estp.2018.5.047

[ref62] Zakaria A. Nordin N. M. (2020). Attitude and self-efficacy and its relationships with entrepreneur intention Among undergraduate students. Int. J. Acad. Res. Busi. Social Sci 10, 145–158. doi: 10.6007/IJARBSS/v10-i14/7684

